# The complete mitochondrial genome of a forensic potential wasp, *Vespa auraria* (Smith)

**DOI:** 10.1080/23802359.2020.1824594

**Published:** 2020-09-29

**Authors:** ZiChao Liu, HongMei Tang, Lei Tong, Xingzhu Liu, FanMing Meng

**Affiliations:** a Engineering Research Center for Exploitation and Utilization of Leech Resources in Universities of Yunnan Province, School of Agronomy and Life Sciences, Kunming University, Kunming, China; b Pu'er Health School of Yunnan Province, Pu'er, China; c Department of Forensic Science, School of Basic Medical Sciences, Central South University, Changsha, China

**Keywords:** Mitogenome, Vespidae, wasp, forensic entomology, *Vespa auraria*

## Abstract

*Vespa auraria* (Smith) is a common wasp species which is broadly distributed in south Asia. This species was spotted in filed forensic studies and disrupted the insect succession on the decaying corpse. Complete mitochondrial genome of *V. auraria* was presented here for its forensically potential influence. The mitogenome was assemblied to 18613 bp in length. The nucleotide composition of present mitogenome was calculated (A: 40.1%, G: 5.5%, T: 41.7%, C: 12.7%). Gene annotation analysis found 22 tRNA, 2 rRNA, and 13 protein-coding genes (PCGs). All the tRNA sequences could be folded into the typical structure of clover-leaf with the excepstion of tRNA-Ser (AGN). Phylogenetic development was analyzed based on *V. auraria* with other species of Hymenoptera.

Species of wasps represent a large group of Hymenoptera with strong mandibles and predate on other insect species or invertebrates (Carpenter 1982). Wasps were also commonly spotted on decaying corpse. According to Catts and Goff (1992), wasps, ants, and other predatory beetles on the corpse could feed on both the corpse and associated fauna, and obviously affect the rates of decomposition by consuming those necrophagous maggots (Richards 1987; Catts and Goff 1992). *Vespa auraria* (Vespidae) is a middle size wasp with typical social behavior (Mattu and Sharma 2017). It could be attracted by a decaying corpse and feed on corpse and insects both. Wasps not only predate on maggots, but adult flies either. When wasps appear on corpse, adult necrophagous flies will escape from the corpse which will disrupt the oviposition of flies on corpse (Goff and Odom 1987). The feeding activities of insects and larger vertebrate scavengers could alter the remains and may leave artifacts trauma that is difficult to be interpreted (Byrd and Castner 2010). Ants, roaches, bees, and wasps produce patterns on human skin that are often confused with perimortem trauma by some investigators (Komar and Beattie 1998; Parker et al. 2010).

Considering the potentially forensic value of *V. auraria*, here we present the complete mitochondrial genome sequence of *V. auraria* (GenBank: MT137096). Voucher specimen used in this study was assigned with a unique code (CUS-MG20200701-002) and deposited in forensic insect herbarium of Department of Forensic Science, Central South University.

The female *V. auraria* samples were captured in Ganchong village, Xiaojie Town, Lufeng County, Yunnan Province, China (N25°05′; E102°02′). The samples were deposited into pure ethyl alcohol and stored under −80 °C before sequencing. The raw DNA materials were extracted from the thorax muscle of *V. auraria*. A total of 12 overlapped PCR primers were designed to amplify the complete mitochondrial genome of *V. auraria*. The procedures and methods of amplification were listed as following: the initial denaturation step was performed at 94 °C for 4 min, followed by 35 cycles reaction of 30 s at 94 °C, annealing step at 50 °C for 30 s, elongation for 1 min at 72 °C, and the final elongation step for 10 min at 72 °C. Then the product of amplification was collected for sequencing. The libraries were constructed with TruSeq Nano DNA LT Sample Preparation Kit (Illumina, San Diego, CA).

The libraries were sequenced on the Illumina HiSeq X Ten platform (Illumina Inc., San Diego, CA) and 150 bp paired-end reads were generated. The reads were assembled into contigs and constructed into the complete mitochondrial genome according to sequence overlapping, and annotated using Geneious (v7.1.4). The online version of ORFfinder was used to confirm the open reading frames feature structure of PCGs. The annotation was improved with Mitochondrial Genome annotation (MITOS) webserver, and the secondary structures of tRNA genes were analyzed by comparing with the nucleotide sequences of other insect tRNA sequences.

Maximum likelihood analysis was conducted based on mitochondrial coding genes of close related species using neighbor-joining methods. *Parapolybia crocea* was used as an outgroup (Figure 1). The result of phylogenetic development was consistent with the previous report (Zhang et al. 2018).

**Figure 1.**
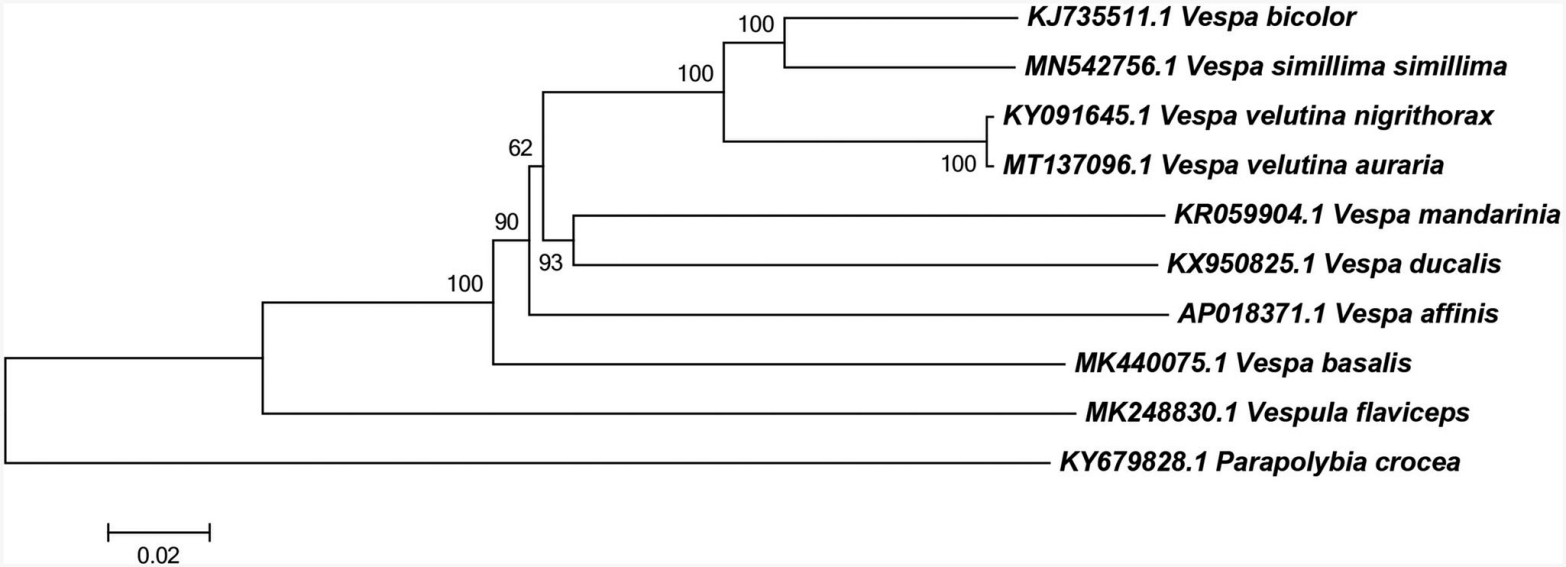


## Data Availability

The data that support the findings of this study are openly available in [Genbank] at [https://www.ncbi.nlm.nih.gov/nuccore/MT137096]
